# Comparative “-omics” in *Mycoplasma pneumoniae* Clinical Isolates Reveals Key Virulence Factors

**DOI:** 10.1371/journal.pone.0137354

**Published:** 2015-09-03

**Authors:** Maria Lluch-Senar, Luca Cozzuto, Jaime Cano, Javier Delgado, Verónica Llórens-Rico, Sabine Pereyre, Cécile Bebear, Luis Serrano

**Affiliations:** 1 EMBL/CRG Systems Biology Research Unit, Centre for Genomic Regulation (CRG), Dr. Aiguader 88, Barcelona, Spain; 2 Universitat Pompeu Fabra (UPF), Dr. Aiguader 88, Barcelona, Spain; 3 Bioinformatics Unit, Centre for Genomic Regulation (CRG) and UPF, Dr. Aiguader 88, Barcelona, Spain; 4 Univ. Bordeaux, INRA, USC-EA3671 Mycoplasmal and Chlamydial Infections in Humans, Bordeaux, France; 5 Bacteriology department, Bordeaux University Hospital, Bordeaux, France; 6 Institució Catalana de Recerca i Estudis Avançats (ICREA), Pg. Lluis Companys 23, Barcelona, Spain; Miami University, UNITED STATES

## Abstract

The human respiratory tract pathogen *M*. *pneumoniae* is one of the best characterized minimal bacterium. Until now, two main groups of clinical isolates of this bacterium have been described (types 1 and 2), differing in the sequence of the P1 adhesin gene. Here, we have sequenced the genomes of 23 clinical isolates of *M*. *pneumoniae*. Studying SNPs, non-synonymous mutations, indels and genome rearrangements of these 23 strains and 4 previously sequenced ones, has revealed new subclasses in the two main groups, some of them being associated with the country of isolation. Integrative analysis of *in vitro* gene essentiality and mutation rates enabled the identification of several putative virulence factors and antigenic proteins; revealing recombination machinery, glycerol metabolism and peroxide production as possible factors in the genetics and physiology of these pathogenic strains. Additionally, the transcriptomes and proteomes of two representative strains, one from each of the two main groups, have been characterized to evaluate the impact of mutations on RNA and proteins levels. This study has revealed that type 2 strains show higher expression levels of CARDS toxin, a protein recently shown to be one of the major factors of inflammation. Thus, we propose that type 2 strains could be more toxigenic than type 1 strains of *M*. *pneumoniae*.

## Introduction

*Mycoplasma pneumoniae* is one of the smallest bacteria that can be grown in axenic culture and it is a frequent agent of community acquired pneumonia in humans, as well as a causative agent of severe extra-pulmonary complications [1–3]. This bacterium is increasingly appreciated for its role in the etiology of reactive airway diseases, such as asthma and adult respiratory distress syndrome (ARDS) [4–6]. In 2005, a toxin encoded by *mpn372*, and termed the Community-Acquired Respiratory Distress Syndrome (CARDS) toxin, was identified as one of the major virulence factors with both monoADP ribosyltransferase (mART) and vacuolating activities [7–9]. The gene encoding the toxin and its promoter were analyzed and it was found that its mRNA levels increase substantially during infection of mammalian cells [10]. Also, during the infection process in mice differences in CARDS levels have been reported for different strains thereby suggesting that CARDS toxin concentrations could be linked to the ability of specific *M*. *pneumoniae* strains to colonize, replicate and persist [11]. The broad spectrum of clinical manifestations [1, 2], its ability to evade the immune system [12] together with a long latency period, are key issues that have hindered the comprehensive understanding of the *M*. *pneumoniae* infection processes.

Epidemics of *M*. *pneumoniae* infection have been spreading worldwide since 2010 [13–18] and they can occur every three to seven years [19, 20]. To identify the source of an epidemic outbreak and decide on treatment, it is important to classify the pathogenic strains rapidly and accurately. There are two main strain types of this bacterium (types 1 and 2), which differ in the sequence of the P1 adhesin gene that is involved in cytoadherence and pathogenicity [21–23]. Clinical analysis of *M*. *pneumoniae* indicates that the prevalence of type 1 and 2 strains seems to shift in subsequent epidemic peaks [24, 25]. In addition, macrolide resistance in *M*. *pneumoniae* was associated with mutations in domain V of 23S rRNA [26]. Variants of each type [20, 27–30] have been described with the differences being localized within two regions, RepMP4 and RepMP2/3, of the P1 adhesin gene [22, 31]. These regions in fact correspond to repetitive sequences of the genome [32]. Additionally by multiple-locus variable-number tandem-repeat (VNTR) analysis (MLVA) of five VNTR loci, namely *mpn1* and *mpn13–16*, 265 *M*. *pneumoniae* strains were classified in 26 MLVA types [33]. Up to date, 60 different MLVA types have been reported [34–36] [37]. However, due to the lack of stability of *mpn1*, the most discriminant marker [34], an amended MLVA nomenclature system based on the four remaining VNTR loci was proposed [37].

Clinical analysis of the infection severity of type 1 and type 2 has failed to reveal an association with the strain genotype [38]. However, there are reports claiming that both types differ in their ability to form microfilms [39]. For example, the strain UAB PO1 (type 2) forms robust biofilms while M129 (type 1) biofilms are weaker [39]. Both strains do produce GlcNAc, however, in M129 this polymer is not tightly attached to the cell surface. Aside from this, significant differences in sequence were found throughout the genome [39].

As discussed above, so far, a comparison of only a few genes and intergenic regions was predominately used to classify the different clinical isolates of *M pneumoniae*. Therefore, it is unclear as to what other differences exist in their genomes, and if these could have any effect on pathogenesis, response to antibiotics, and/or the immune system. Recently, apart from the classical M129 strain (type 1; NCBI reference sequence: NC_000912.1), other strains have been fully sequenced and annotated: M129-B7 (type 1; NCBI reference sequence: NC_020076.1; CP003913.1), FH (type 2; NCBI reference sequence: NC_017504.1; CP002077.1), 309 (type 2a; NCBI reference sequence: NC_016807.1), and UAB PO1 (type 2; [39]), but no detailed comparative analysis of their genome differences has been done.

In the past few years, the *M*. *pneumoniae* type 1 M129 strain has been extensively characterized by diverse ‘-OMICSs’ studies. Thus, we have a full analysis of all its transcriptome [40, 41], metabolome [40, 42], proteome [40], as well as protein modifications [43] and proteins half-lives [44]. Regarding the FH strain, considered as reference for tyep2, has shown a certain capacity for homologous recombination [45], as well as, it has been analyzed at morphological level [46].

Although multifaceted approaches for the characterization of *M*. *pneumoniae* and its associated diseases have augmented, most mycoplasma infections in clinical settings do not have a microbiological diagnosis. Thus, serological tests are the only means by which *M*. *pneumoniae* infections are diagnosed on a wide scale. Due to this method having a number of limitations, nucleic acid amplification tests (NAATs) have been developed and are now widely used for the diagnosis of *M*. *pneumoniae* infections. Additionally, the development of a safe vaccine that offers protective immunity might also go a long way towards reducing the extent of *M*. *pneumoniae* infections. Identification of virulence factors is crucial for gaining insight into the pathogenesis of *M*. *pneumoniae*, designing new NAAT diagnosis targets and efficient vaccines. Herein, we identify these factors by doing a comparative genome study across 27 *M*. *pneumoniae* clinical isolates. Genome sequencing combined with a recent essentiality study, performed in *M*. *pneumoniae* M129 strain [47], led to the identification of antigenic proteins as well as, virulence factors. Furthermore, transcriptome and proteome analysis of four representative strains of both types, revealed higher amounts of CARDS toxin in type 2 strains. This result in conjunction with the ability of type 2 strains to form stronger biofilms [39], suggests that type 2 strains could be more virulent that type 1.

## Results and Discussion

### Genome sequencing of *M*. *pneumoniae* strains

**Fig 1:**
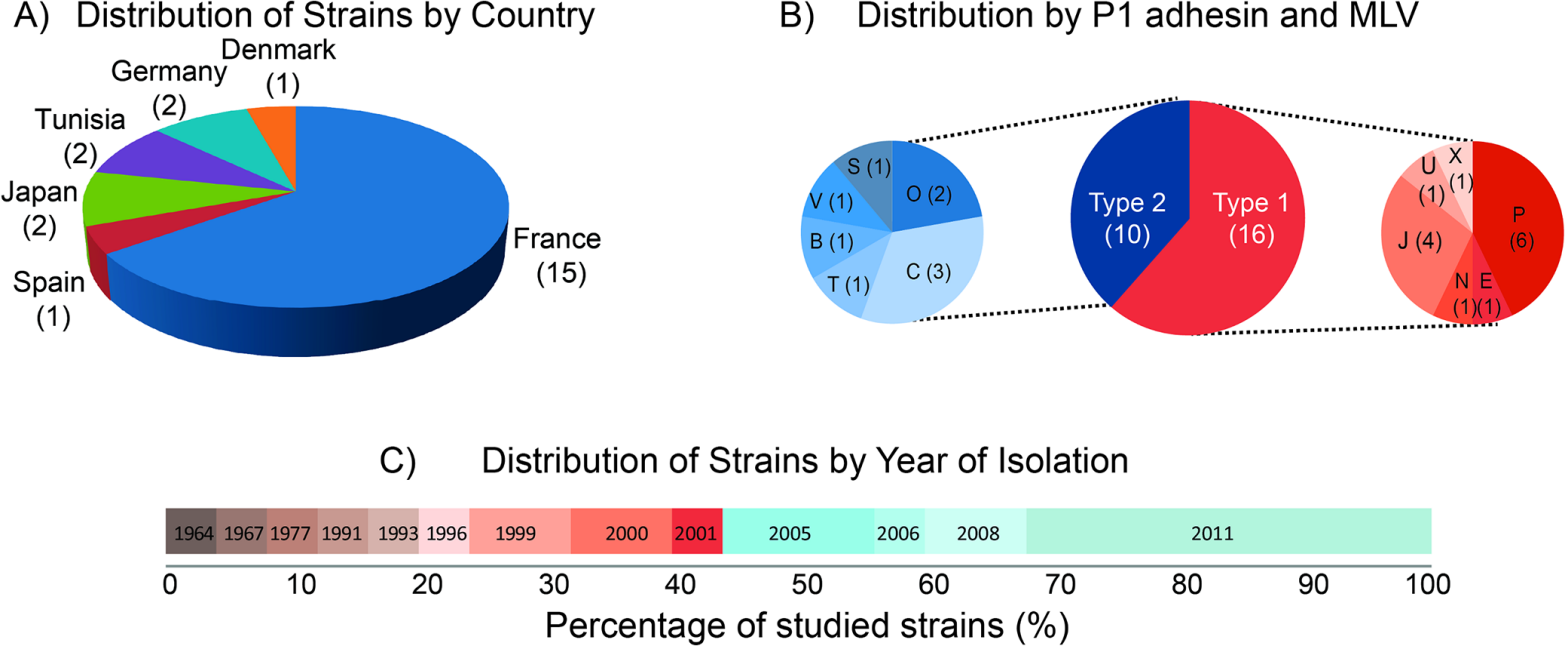
Features of the 22 *M*. *pneumoniae* clinical isolates. A) Distribution of different strains by countries. B) Distribution of strains considering the typing described in S2 Table. C) Distribution of strains by year of isolation.

We studied 23 *M*. *pneumoniae* strains isolated at different years from patients of six different countries. These strains have been classified by restriction fragment length polymorphism (RFLP) analysis of PCR products of the *M*. *pneumoniae* adhesin P1 gene and by MLVA types (from years: 1964 to 2011; Fig 1; S1 Table). Genomic DNAs of the strains were sequenced by Illumina GAII. We obtained ~six million reads per sequenced genome, representing average genome coverage of ~1500 (2x100 per read/genome size). After filtering the initial raw reads, the genomes were mapped against the reference genomes: *M*. *pneumoniae* M129 and *M*. *pneumoniae* FH strains (NCBI Reference Sequences: NC_000912.1 and NC_017504.1; S1 Table). As shown in S2 Table, the fraction of reads mapping to the reference genomes is always higher than 98% (data not shown), and their orientation preserved in more than 96.5% for M129 and 95.9% for FH of read pairs, thus suggesting that within the strains different degrees of rearrangements exist. We assembled the filtered reads (see methods section), obtaining a number of scaffolds for every strain, ranging from 19 up until 45, with an N50 bigger than 80 kb and an E-size bigger than 100 kb (S2 Table).

### Strain classification

**Fig 2:**
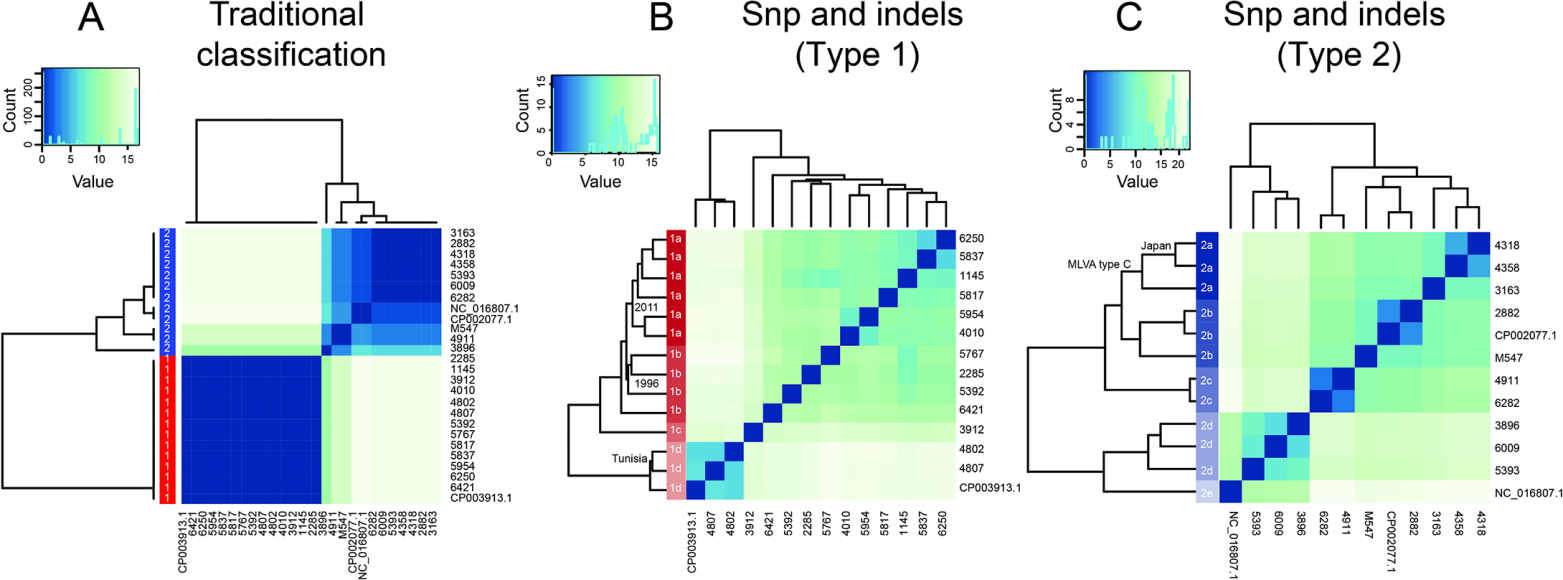
Clustering of different *M*. *pneumoniae* strains. Clustering has been performed by considering the 27 sequenced strains (23 from the current study and 4 previously sequenced ones: NC_000912.1, CP003913.1, NC_016807.1 and CP002077.1). A) The criteria used for the classification are the mutations described in S3 Table; corresponding to the mutations usually considered for typing. B) Classification considering single nucleotide deletions (indels) and SNPs (synonymous and non-synonymous mutations) for type 1 strains using as reference M129. C) Clustering of type 2 strains by SNPs and Indels, considering FH strain as reference.

We did a comparative study across the 27 sequenced strains (23 from the current study and four already sequenced ones: NC_000912.1, CP003913.1, NC_016807.1 and CP002077.1). These strains were previously classified based on the sequence of the P1 adhesin gene as Type 1 (1145, 2285, 3912, 4010, 4802, 4807, 5767, 5817, 5837, 5954, 6250, 6282, 6421), Type 2 (2882, 3163, 4318, 4358, 4911, M547), Type 2a (5393, 6009), and new type (3896). A first classification considering the six SNPs commonly used for type 1 and type 2 taxonomy was performed (Fig 2A; S3 Table; [29]). Two main groups were found comprising 14 and 12 strains, respectively. The biggest cluster included the type 1 cataloged strain CP003913.1 suggesting that these 14 strains were type 1. The second cluster included the sequenced and classified type 2 strains NC_016807.1 and CP002077.1. Strain 6282, previously considered by P1 adhesin typing as type 1 [29], is classified as type 2 in the current study.

Taking advantage of the complete genome sequencing, a clustering was performed by considering a binary matrix (1,0) for the presence/absence of mutations in a particular strain for all genome sequences (Fig 2B and 2C; S4 Table). For this study, NC_000912.1 (MPN129) and CP002077.1 (FH) were used as references for determining the SNPs, indels and non-sysnonymous mutations in type 1 and type 2 strains, respectively. Four subtypes were observed in the type 1 group (1a-2d; Fig 2B), and five in the type 2 group (2a-2d; Fig 2C). Interestingly, most of the subtype 1a strains were isolated in 2011 whilst subtype 1b strains were mostly isolated before 2000 (S1 Table). Strain 3912 isolated from France in 2005 is classified as a new subtype, is considered a divergent type 1c strain. The new subcluster in type 1 (type 1d) comprises strains isolated from Tunisia and the previously sequenced strain CP003913.1. Additionally, in the type 2, we found a new subcluster (type 2a) comprising three strains isolated in the early 2000 and belonging to the MLVA type C. Also, 2 of them were isolated in Japan (4318 and 4358; Fig 2C). These analyses confirmed that genome sequencing and comparison between different strains renders a more accurate classification that could enhance the finding of genomic properties associated to pathogenicity aspects. From now on in the text we will use our classification based on SNPs and indels for the 27 strains.

We also analyzed the impact of genome rearrangements and deletions on strain classification (S1 Fig; S6 Table). Even though we were able to find the two main groups, the classification was more diffuse, indicating that the frequency of chromosomal rearrangements is higher than the frequency of SNPs and indels.

### Antigenic variation strategies: mutation rates and genome rearrangements in adhesin P1 types

**Fig 3:**
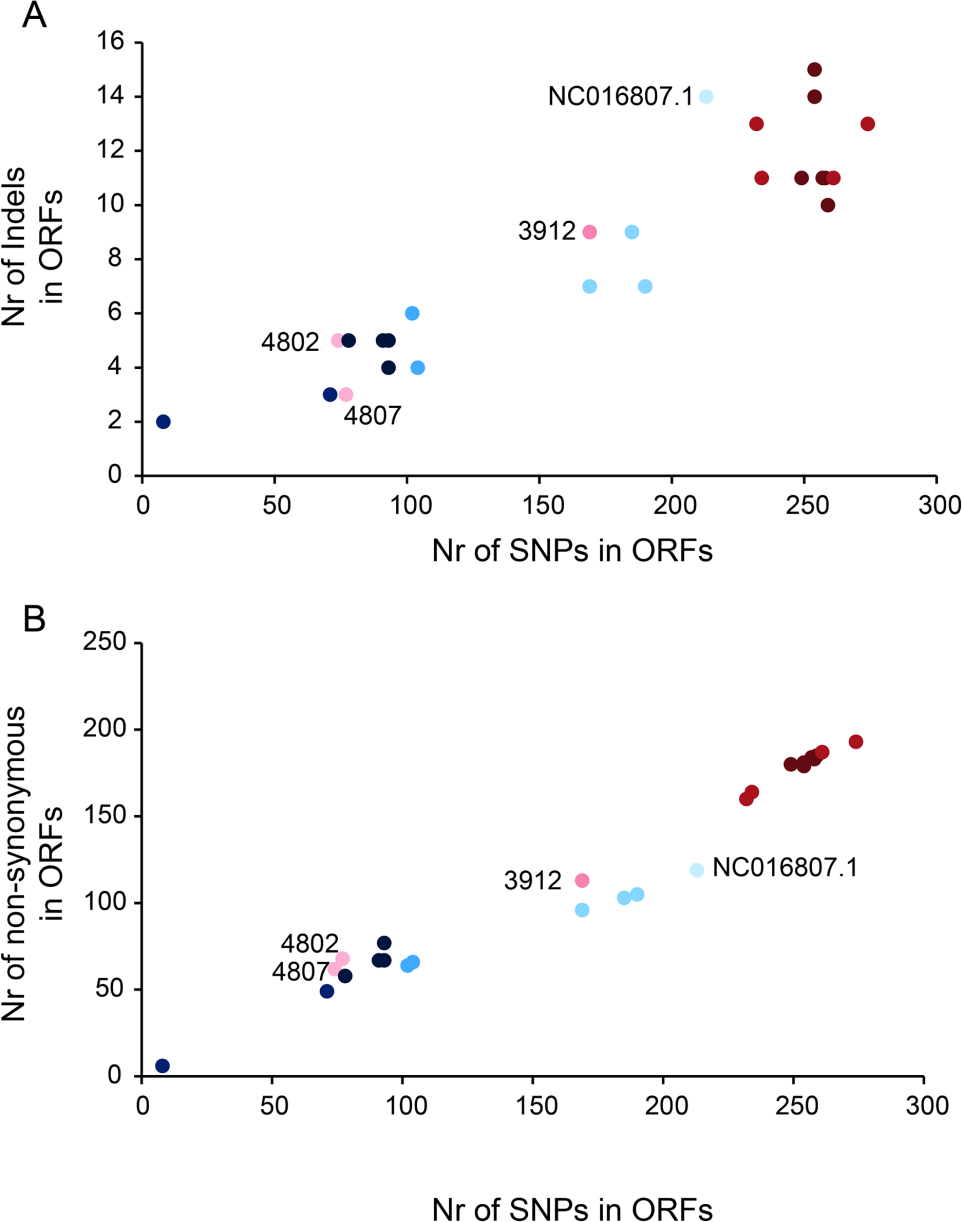
Study of indels, SNPs and non-synonymous mutations in ORFs and ncRNAs. A) Correlation of numbers of SNPs and indels for the ORFs of *M*. *pneumoniae* genome. B) Representation of numbers of SNPs versus non-synonymous mutations in ORFs. In all panels, red and blue dots indicate type 1 and type 2 strains, respectively and the color gradient the subtypes as shown in Fig 2B.

Analysis of the SNPs vs indels and SNPs vs non-synonymous mutation numbers in each strain revealed that these values have a high correlation (strains with a high SNPs number also show a high indel and non-synonymos mutation numbers; r = 0,92 and r = 0.98, respectively; Fig 3A and 3B; S4 Table). Indels, non-synonymous and SNPs numbers allowed the separation of the different strains into subgroups (Fig 2B and 2C). Interestingly, three main subtypes were observed, showing in the type 1 strains more genomic changes. The 4802 and 4807 strains from Tunisia (subtype 1d) show similar mutation numbers to type 2a-b strains and 3912 (subtype 1c) show similar mutation numbers to strains of subtype 2d. Additionally, already classified and divergent NC016807.1 strain (Fig 2C) shows a mutation number similar to type 1 strains.

The study of functional enrichment in COG categories for ORFs with SNPs indels and non-synonymous mutations revealed an enrichment for genes encoding proteins involved in cell envelope biogenesis in type 1 stains (Fisher’s tests in S7 Table), and virulence genes were also enriched in indels in type 1 stains (S7 Table). In type 2 strains, enrichment in indels and non-synonymous mutations was found in genes encoding proteins involved in cell envelope biogenesis, also category of virulence genes was enriched in indels (S7 Table). These results agree with the proposition that proteins involved in adhesion and virulence that have multiple copies in the genome, experience frequent reorganization by recombination during the infection process [48]. Taking advantage of the transcriptome information of *M*. *pneumoniae* M129 [41], we have compared SNPs and indels rates in coding (ORFs, 737) and non-coding regions (ncRNAs, 311) and non-transcriptionally active regions, in type 1 strains. SNPs and indel numbers in ORFs and ncRNAs showed a linear correlation (r = 0.97 and r = 0.95, respectively; S4 Table) and SNPs in regions that are non-transcriptionally active also correlate with those in ORFs and ncRNAs (r = 0,94; S5 Table) suggesting that mutations affect similarly to all genomic regions.

In total, the number of ORFs showing SNPs was 240 in types 1 and 2, respectively (123 common ORFs) with 182 and 170 of these harboring non-synonymous mutations in type1 and 2, respectively (75 common ORFs) (S7 Table). Only 26 ORFs and 21 ORFs, mainly encoding for hypothetical proteins, showed frameshifts in the 1 and 2 types, respectively (S8 Table). The study of functional enrichment in COG categories by using a Fisher’s test revealed that ORFs showing frameshift mutations encode for proteins involved in cell envelope biogenesis in both strain types (odd ratio = 3.9 and p-value = 0.001 for type 1 and odd ratio = 3.7 and p-value = 0.009 for type 2).

### Mutation numbers and *in vitro* essentiality: putative virulence factors

**Fig 4:**
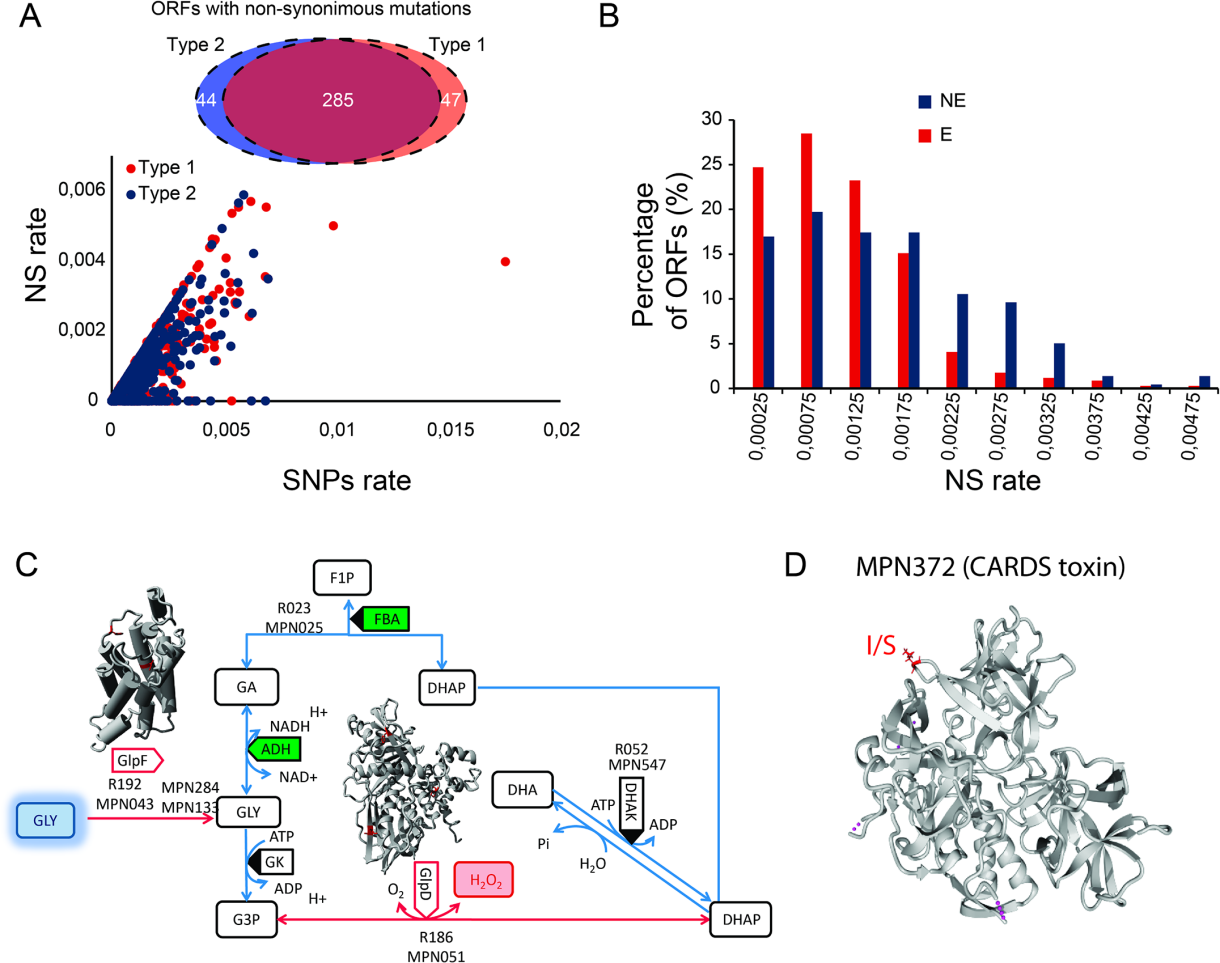
Study of common mutations in type 1 and type 2 strains. A) The Venn diagram indicates the number of genes showing non-synonymous mutation in type 1 strains (red circle) and in type 2 strains (blue circle). The dots in the graph indicate the SNPs versus non-synonymous (NS) rates for all the genes in type 1 (red dots) and type 2 (blue dots). B) The histogram represents the number of genes versus densities of non-synonymous mutations in type 2 strains for essential ORFs (E, red bars) and non-essential ORFs (NE, blue bars). Essentiality categories were described in Lluch-Senar et al [47]. C) Representation of the glycerol pathway in *M*. *pneumoniae* metabolism. The protein structures of the orthologous GlpF and GlpD proteins of *Escherichia coli* [52, 53] are represented in grey and the corresponding mutated residues in *M*. *pneumoniae* are represented in red. Red arrows represent the reactions where both proteins are involved. D) Protein structure of *M*. *pneumoniae* CARDs toxin [56]. The mutated amino acid is represented in red in the protein structure.

Essential genes for *in vivo* survival, virulence and pathogenicity are expected to have mainly silent mutations that do not change the amino acid sequence of the protein, or non-synonymous mutations that do not affect protein function. Those genes having a significant number of non-synonymous mutations could be either non-essential for infection, or under strong selection by the immune system, but likely quite essential for successful *in vivo* survival. To identify genes essential for infection and pathogenicity, we compare the SNPs rate versus the non-synonymous rate for the 429 and 432 ORFs showing mutations in the type 1 (using as reference FH strain) and type 2 (using as reference M129), respectively (S9 Table; Fig 4A) Interestingly, the distribution of SNPs versus non-synonymous rates reveals three groups of genes that probably reflect essentiality profiles. It is important to note that those genes with high SNPs rates but low non-synonymous rates could be essential for infection. In total, 100 ORFs showed SNPs but no non-synonymous mutations in the 1 and 2 type strains, suggesting that they cannot tolerate aa changes. Interestingly, 66 ORFs were common to both strain types and others were specific to each type (S9 and S10 Tables). Through transposon mutagenesis we found that in the genome of *M*. *pneumoniae* 49.3% of ORFs are essential (E, 363 out 737 ORFs), 37.4% non-essential (NE, 276 out 737 ORFs) and 13.3% fitness (F, 98 out 737) for *in vitro* growth [47]. Studying the distribution of non-synonymous densities in both strain types in E and NE ORFs (after discarding ORFs not having mutations) revealed two different distributions (Fig 4B). As expected, NE ORFs show higher rates (>0.002) of non-synonymous mutations (odd ratio: 3.4 p-value of 1.5x10^–7^after applying the Fisher’s test). Although there is significant overlap between the two distributions, we speculate that the ORFs classified *in vitro* as NE, could become essential during infection, and have a similar non-synonymous mutation rate as the E genes (rate of mutations <0.002; odd ratio: 1.3 p-value of 0.03 in E ORFs after applying the Fisher’s test). By considering the distribution of non-synonymous mutations in ORFs (Fig 4B), we found 15 *in vitro* NE ORFs that could be essential genes in all pathogenic strains (NE ORFs with SNPs but no non-synonymous mutations) (S9 Table). Applying the Fisher’s test to functional COG categories revealed an enrichment of genes involved in DNA recombination and repair (Fisher’s test; odd ratio = 3.6 and p-value = 0.02 for COG L; S9 Table). Interestingly, *mpn535* and *mpn536* encoding for RuvA and RuvB proteins, respectively, are NE *in vitro* yet became essential in the infection process. In *M*. *genitalium*, the deletion of *ruvA* and *ruvB* genes impaires its ability to generate antigenic variation of the MgpB and MgpC adhesins [49]. Additionaly, RuvA and RuvB have been described to have a key role in the infectivity of *Borrelia bugdorferi* by promoting the recombination of the *VlsE*, a gene which encodes a surface-exposed lipoprotein [50]. All these data taken together suggest that recombination by RuvAB could be an essential mechanism for the pathogenicity of *M*. *pneumoniae*.

We evaluated the antigenic variation by estimating the non-synonymous density in all ORFs and calculating the probabilities of finding the observed number of mutations (or higher) in a given gene, considering its size. Our null hypothesis is that these probabilities follow a binomial distribution with a probability of success equal to the density of mutations averaged across all the ORFs (S9 Table). To look for genes that have a higher than expected non-synonymous mutation rate we used a probability value lower than 0.03 and a p-value<0.05, and were able to identify 73 putative antigenic genes common to both strain types (28 E, 10 F and 35 NE; S9 Table).

The Fisher’s test in COG categories revealed an enrichment in M category, corresponding to proteins involved in cell envelope biogenesis (by Fisher’s test: odd ratio = 2.42 and p-value = 0.003; S9 Table). Interestingly, *glpF*, encoding for the glycerol uptake facilitator, although being essential for *in vitro* growth, shows a proportionally large number of non-synonymous mutations (Fig 4C). This suggests that the GlpF protein could be a target of the host’s immune system. It is tempting to speculate that by mutating the transporter the bacteria fools the immune system, and thereby preserves this glycerol pathway that may be essential for pathogenicity and infection [51]. In fact, another gene that shows an even higher mutation rate is *glpD*, which encodes for the glycerol-3-phospate dehydrogenase, an enzyme also involved in glycerol metabolism as well as in the production of hydrogen peroxide [52, 53]. This enzyme is already described as one of the major virulence factors of *M*. *pneumoniae* [51] (Fig 4C). However, recent findings with *M*. *gallisepticum* describe that glycerol metabolism genes do not seem to be important for pathogenicity in the natural host [54]. Thus, the high rate of non-synonymous mutations in glycerol metabolism genes might also suggest that these are not important for virulence in the human host.

### Transcriptome and proteome to evaluate the impact of mutations on expression

Mutations in promoters, or regulatory regions, can alter the levels of transcripts resulting in a variation at the protein level. Similarly, mutations in coding regions can affect protein stability and thus protein copy number. To evaluate the impact of genome modification on the transcriptome and proteome we selected representative strains of the main types and subtypes (type 1a: 5954 and 5817; type 2a: 2882 and 6009) and studied them by deep-sequencing (RNAseq; S11 Table) and mass spectroscopy (MS; S12 and S13 Tables), respectively.

Significant differences in RNA levels from two biological replicates (fold changes with p-value<0.05) between the strain types were observed for 69 ORFs (49 coding regions and 20 non-coding RNAs; S11 Table). The study of functional enrichment by COG categories revealed that ORFs involved in cell envelope biogenesis are more highly expressed in type 2 compared to type 1 strains (Fisher’s test: odd ratio = 5 and p-value = 0.0003; S11 Table). Five out of 12 ORFs from this category were not found in type 1 strains nor the protein detected by MS, thereby suggesting that they are indeed not expressed. This study using COG categories revealed differences in expression for genes involved in gliding motility, being less expressed in type 2 strains (Fisher’s test: odd ratio = 6.6 and p-value = 0.003; S11 Table).

We also did a proteome analysis (two independent experiments, comprising in total three biological replicates and six technical replicates; S12 Table) to identify mutations that could affect protein half-life or translation efficiency. MS is less sensitive than RNAseq, and thus low abundant proteins or proteins without unique peptides are difficult to detected (63% of proteins have been detected) (S12 and S13 Tables). However, we were capable of identifying three proteins: MPN388 (hypothetical protein), MPN372 (CARDS toxin) and MPN115 (InfC), which showed significant reproducible differences in relative protein abundance upon comparing the proteomes of type 1a and type 2a strains (by applying paired t-test; S12 Table). As mentioned above, *mpn372* encodes for CARDS toxin [55, 56]. In all type 2 strains we found a T/G mutation at position 1112 of the *mpn372* gene (I371S in the corresponding protein; Fig 4D). A FoldX study of protein structure [57] revealed that this mutation is not affecting protein stability (difference in energy = 0.116617 Kcal/mol). Being as the transcriptome analysis did not reveal significant differences in *mpn372’s* expression, the variation in protein level likely stems from unknown factors. Since the mutation is at the protein surface (Fig 4D), it could potentially affect the interaction with clathrin [58], and as a consequence its import into the target human cells. In contrast, this mutation could have the opposite effect and make type 2 strains less virulent by inhibiting secretion through interactions with clathrin or other mechanisms. Type 2 strains should then produce more of the CARDS toxin protein in order to display the same level of virulence as type 1 strains because of the mutation.

In conclusion, through genome sequencing it was possible to identify all SNPs, non-synonymous mutations, indels, and rearrangements in the genome of different *M*. *pneumoniae* strains, allowing us to further refine the typing and reveal subclasses within the two main groups. We find that SNPs and indels correlate for all genomic regions (transcriptionally active and intergenic) across all strains, and that type 1 strains have higher non-synonymous mutation rates compared with our reference M129 (type 1). However, the total number of non-synonymous mutations and SNPs across all strains is not high (max 274 and 193 in ORFs, respectively), indicating that *M*. *pneumoniae*, although having a small genome with reduced DNA repair machinery, does not evolve fast. This is not the case for deletions and rearrangements for which we see differences among strains in the same subtype, validating the hypothesis that recombination between repetitive elements allow *M*. *pneumoniae* to evade the immune system [48].

Integrative analysis of *in vitro* gene essentiality [47] and mutation rates led to the identification of some putative virulence factors and antigenic proteins, confirming that glycerol metabolism and peroxide production are both important factors in the physiology of these pathogenic strains. Additionally, transcriptomic and proteomic data helped in characterizing the impact of mutations on the levels of RNA and proteins. As discussed above, we find a surprising conservation of RNA and protein expression levels, with only 47 RNAs and three proteins changing significantly in abundance between type 1a and 2a strains. This result suggests that *M*. *pneumoniae* is very well adapted to its host. An interesting finding is that type 2a strains have higher levels of the CARDS toxin showing that this toxin is differently affected in the two types Since this protein was shown to induce a strong immune response [59], and it was described that type 2 strains make biofilms [39], these data suggest that type 2 strains could be more toxigenic than type 1. However, further information is needed to confirm the virulence level of CARDS toxin between both types of *M*. *pneumoniae* strains.

## Materials and Methods

### *M*. *pneumoniae* growth conditions

*M*. *pneumoniae* was grown in 50 mL of modified Hayflick medium supplemented with glucose at 37°C as previously described [60].

### Genomic DNA library preparations

Genomic DNA was collected using the Illustrabacteria genomic Kit (GE Healthcare) and sheared to 100 bp fragments using a Covaris S2 device. Paired-end Illumina libraries were created as described by Bentley et al. [61] and the size selected to be between 200 and 400 bp. The resulting libraries were quantified on an Agilent Bioanalyzer chip (Agilent Technologies). Double-stranded templates were cluster amplified and sequenced on an Illumina GAII. The raw data of DNAseq was submitted to have been upload to SRA with the accession number SRP061659. The sequences of the assembled genomes have been deposited at DDBJ/EMBL/GenBank with the indicated accession names: LHPO00000000, LHPP00000000, LHPQ00000000, LHPR00000000, LHPS00000000, LHPU00000000, LHPO00000000, LHPV00000000, LHPW00000000, LHPX00000000, LHPZ00000000, LHPO00000000, LHQA00000000, LHQB00000000, LHQC00000000, LHQE00000000, LHQA00000000, LHQF00000000, LHQG00000000, LHQH00000000, LHQI00000000, LHQJ00000000 and LHQK00000000.

### De novo assembly, variant calling and bioinformatics analysis

Raw reads were analyzed by using the FastQC tool (website: http://www.bioinformatics.babraham.ac.uk/projects/fastqc/ Key: citeulike:11583827) for assessing the quality and the presence of adapters, and then filtered according to the following criteria: i) reads containing the adapter were removed by the program tagdust [62]; ii) unchaste reads were removed; iii) the latest 5 bases at the 3’ end were trimmed; iv) mean phred quality of the trimmed read > = 20; v) a maximum of two low quality bases (phred quality<5) was allowed.

Filtered reads corresponding to a genome coverage of 150X were assembled by using the Abyss program [63]. Briefly, reads were fragmented in k-mers and connected depending on whether they shared the same sequence of length k-1. Ambiguities were solved by counting the number of reads connecting different edges, and scaffolds were produced by considering the mate pairs information. For each assembly we calculated the N-50 and the E-size [64] by using the abyss-fac tool from the Abyss package and we mapped back the original reads to the scaffolds by using bowtie2 with options—very-sensitive-X 1000 (version 2.2.5) [65] to estimate the quality of the assembly (S2 Table; column “remapping”).

Subsequently, the resulting scaffolds were mapped to the reference genomes (M129 and FH) by using bwa mem tool (version 0.7.12-r1039) [66] [67]. Variant calling was performed by using samtools and bcftools (version 0.1.18) [68] on both alignments obtained by mapping scaffolds and reads the references. Variations (indels and SNPs) were kept only if they are found from both procedures.

Variations were annotated using the Variant Effect Predictor tool [69] with annotations from Ensembl Bacteria database for FH strain (GCA_000143945.1) and HomoConTrans19 for M129 [47].

Heat-maps were generated by using the heatmap.2 function in the gplots package [70]. A binary matrix indicating the presence or absence of each variation in given samples is used to calculate the Euclidean distance between the samples and then fed to the heatmap.2 function for clustering and displaying.

Genes from M129 and FH strains were considered orthologous when detected by the ProteinOrtho tool [71] imposing 50% of coverage and identity and using the synteny option.

### Sample Preparation for LC–MS/MS

*M*. *pneumoniae* type 1 (5954 and 5817) and type 2 (2882 and 6009) strains were grown for 96 h at 37°C. Then the medium was removed and cells were washed twice with PBS. Total protein extracts were obtained by lysing the cells with 200 μl of lysis buffer (4% SDS, 0.1M DTT and 0.1M Hepes). The total protein extracts of two biological replicates were analyzed by MS.

Each fraction (quantities ranging from 20 to 486 μg) was digested in solution with trypsin. Briefly, samples were dissolved in 6 M urea, reduced with 10 mM dithiothreitol (37°C, 60 min), and alkylated with 20 mM iodoacetamide (25°C, 30 min). Samples were diluted 10-fold with 0.2 M NH_4_HCO_3_ before being digested at 37°C overnight with trypsin (ratio protein:enzyme 10:1). Peptides generated upon digestion were desalted, evaporated to dryness and dissolved in 300 μl of 0.1% formic acid. An aliquot of 2.5 μl of each fraction (amounts ranging from 0.17 to 4 μg) was run on an LTQ-Orbitrap Velos (Thermofisher) fitted with a nanospray source (Thermofisher) after a nanoLC separation in an EasyLC system (Proxeon). Peptides were separated in a reverse phase column, 75 μm x 150 mm (Nikkyo Technos Co., Ltd.) with a gradient of 5 to 35% acetonitrile in 0.1% formic acid for 60 min at a flow rate of 0.3 mL/min. The Orbitrap Velos was operated in positive ion mode with the nanospray voltage set at 2.2 kV and its source temperature at 325°C. The instrument was externally calibrated using Ultramark 1621 for the FT mass analyzer and the background polysiloxane ion signal at m/z 445.120025 was used as lock mass. The instrument was operated in data-dependent acquisition (DDA) mode and in all experiments full-MS scans were acquired over a mass range of m/z 350–2000, with detection in the Orbitrap mass analyzer at a resolution setting of 60,000. Fragment ion spectra produced via collision induced dissociation (CID) were acquired in the ion trap mass analyzer. In each cycle of data-dependent analysis, following each survey scan the top twenty most intense ions with multiple charged ions above a threshold ion count of 5000 were selected for fragmentation at a normalized collision energy of 35%. All data were acquired with Xcalibur 2.1 software. In addition, 20 μg of the total extract was digested and desalted and 1 μg of the resulting peptides analyzed on an Orbitrap Velos Pro in the same conditions as the fractions but with a longer gradient (120 min).

A total of three biological replicates were done, as well as two technical replicates for each strain in two independent experiments. The spectra were assigned to peptides by using Mascot and a customized database comprising all the ORFs longer than 19 amino acid. Only the areas of the three best unique peptides were used to estimate the protein amounts. Fold changes were calculated by comparing each strain individually against the others, or grouping by types. The paired T-test was used to find the significant fold changes.

The mass spectrometry proteomics data have been deposited to the ProteomeXchange Consortium [72] via the PRIDE partner repository with the dataset identifier PXD002501

### RNA extractions and sample preparations

After growing *M*. *pneumoniae* for 6h at 37°C, cells were washed twice with PBS and lysed with 700 μl of Qiazol buffer. Then, samples were lysed with 700 μl of Qiazol buffer. RNA extractions were performed by using the miRNeasy mini Kit (Qiagen) following the instructions of the manufacturer. Libraries for RNA-seq were prepared following directional RNA-seq library preparation and sequencing. Briefly, 1 μg of total RNA was fragmented to ~100–150 nt using NEB Next Magnesium RNA Fragmentation Module (ref. E6150S, NEB). Treatments with Antarctic phosphatase (ref. M0289S, NEB) and PNK (ref. M0201S, NEB) were performed in order to make the 5’ and 3’ ends of the RNA available for adapter ligation. Samples were further processed using the TruSeq small RNA Sample Prep Kit (ref. RS-200–0012, Illumina) according to the manufacturer's protocol. In summary, 3’ adapters and subsequently 5’ adapters were ligated to the RNA. cDNA was synthesized using reverse transcriptase (SuperScript II, ref. 18064–014, Invitrogen) and a specific primer (RNA RT Primer) complementary to the 3’ RNA adapter. cDNA was further amplified by PCR using indexed adapters supplied in the kit. Finally, size selection of the libraries was performed using 6% Novex® TBE Gels (ref. EC6265BOX, Life Technologies). Fragments with insert sizes of 100 to 130 bp were cut from the gel, and cDNA was precipitated and eluted in 10 μl of elution buffer. Double-stranded templates were cluster amplified and sequenced on an Illumina HiSeq 2000. The raw data of RNAseq was submited to the GEO database with the accession number GSE71467.The counts per kilobase per million reads (CPKM) value was calculated for each ORF and ncRNA as follows:

$$\text{CPKM}=\frac{\text{counts per gene}}{\left(\text{total counts}/10^{6})\times (\text{gene length}/10^{3}\right)}$$

The CPKM vales were converted to log2 values.

## Supporting Information

S1 Fig Classification considering the genome rearrangements that promote large genome deletions (S6 Table). (TIFF)

S1 Table Features of the sequenced strains. A) Number assigned to the strain. B) Selected category. C) Country where it was isolated. D) Year of isolation. E) Anatomical site from which the strain was obtained. F) Age of the patients from whom the strains were isolated. G) Classification by MLVA type. H) Classification by P1 adhesin. I) Classification considering all SNPs described in S3 Table. J) Classification by indels and SNPs. K) Classification by genome rearrangements.
(XLSX)

S2 Table Results of the genome mapping and assembly. A) Number assigned to the strain. B) Number of reads obtained in the sequencing. C) Percentage of reads mapped in the correct orientation using as reference M129 strain (type 1). D) Percentage of reads mapped in the correct orientation using as reference FH strain (type 2). E) Number of scaffolds in the genome assembly. F), G) and H) The N50, e-size and maximum values obtained in the assembly, respectively. I) Genome sizes determined after the genome assembly. J) Percentage reads mapped in the correct orientation using the assembled scaffolds as reference.
(XLSX)

S3 Table Description of the genomic modifications usually considered for typing of *M*. *penumoniae* strains. (XLSX)

S4 Table Description of mutation rates. In the rows 1 to 29 and columns A to N, the table indicates the number and rates of indels, SNPs, non-synonymous, and frameshift mutations in ORFs and ncRNAs for type 1 and type 2 strains using as reference M129 strain (type 1). In the rows 32 to 60 and columns A to N, the table indicates the number and rates of indels, SNPs, non-synonymous, and frameshift mutations in ORFs and ncRNAs for type 1 and type 2 strains using as reference FH strain (type 2).In the legend assigned to the table is the description of the equations used for rate calculations.
(XLSX)

S5 Table Study of mutation rates in different genomic regions. In column A, names of type 1 strains. Columns B and C the number of SNPs found in regions that are transcriptionally active (ORFs and ncRNAs) and number of SNPs in intergenic, non-transcriptionally active regions. Coluns D and E, densities of SNPs in the different regions calculated by using the formula included in the attached table description in column F.
(XLSX)

S6 Table Deletions found in the genomes of the different sequenced strains. The first row indicates the genomic region that has been lost. The last row indicates the genes comprised by the deletion. (should we move this after S5??)
(XLSX)

S7 Table Description of genes that show SNPs, indels and mutations in the type 1 and type 2 strains. Columns A and B show names for the 567 orthologous genes found in M129 and FH reference strains, respectively (see methods section for their identification). C and D show the gene sizes in the two reference strains. E to G columns show descriptions of gene functionality. The mutations (SNPs, Indels and non-synonymous) have been extracted by considering M129 as reference for type 1 and FH for type 2 strains, respectively. SNPs rates (columns H and K) are calculated by considering the number of mutations in each ORF divided by the number of strains and the gene size for each type (gene size of M129 for type 1 and orthologue gene size FH for type 2). Indels rates (columns I and L) are calculated by considering the number of nucleotide deletions in the ORF divided by the number of strains and the gene size for each type. Non-synonymous rates (columns J and M) are calculated by considering the number of mutations that promote an aa change in the ORF, divided by the number of strains and the gene size of each type. Also, the Fisher’s tests done to study the functional enrichment is COG categories (for ORFs harboring SPNs, indels and non-synonymous mutations) are shown in the two additional tables; for type 1 and type 2 strains, respectively.
(XLSX)

S8 Table Study of the genes that show frameshifts in the sequenced strains. The upper table shows the ORFs with frameshifts, where 0 indicates no frameshift and 1 indicates the presence of frameshift when M129 strain is used as reference. The lower table shows the ORFs with frameshifts, where 0 indicates no frameshift and 1 indicates the presence of frameshift when FH strain is used as reference. Also, the results of the Fisher’s test used to study functional enrichment by COG categories are shown in the additional tables.
(XLSX)

S9 Table Description of common SNPs and non-synonymous rates for each gene. Columns A and B show gene name in M129 and FH strains, respectively. In C and D columns are indicated the gene sizes for M129 and FH strains, respectively. The description of functionality of the different genes is shown in the columns E to H. COGs: A, membrane proteins of unknown function; C, energy production and conversion, coenzyme metabolism; D, cell division and chromosome partitioning; E, amino acid transport and metabolism; F, nucleotide transport and metabolism, coenzyme; G, carbohydrate transport and metabolism; H, coenzyme metabolism; I, lipid metabolism; J, translation, ribosomal structure, and biogenesis; K, transcription; L, DNA replication, recombination, and repair; M, cell envelope biogenesis, outer membrane; N, cell motility and secretion; O, post-translational modification, protein turnover, chaperones; P, inorganic ion transport and metabolism; R, general function prediction only; S, function unknown; T, signal transduction mechanisms; U, intracellular trafficking, secretion, and vesicular transport; and V, defense mechanisms. Gene essentiality (column G) determined by transposon mutagenesis [47]. E, essential; NE, non-essential; NE*, non-essential with repetitive regions; F, fitness. Columns I and J indicate SNPs and non-synonymous rates for type 1 strains, respectively. Same information is shown in columns K and L for type 2 strains. Muatations have been estimated by using as reference FH for type 1 strains and M129 for type 2 strains. In the columns M to P ate the probability and p-value of non-synonymous mutations found for each gene in type 1 and type 2 strains. The null hypothesis is that these probabilities follow a binomial distribution with a probability of success equal to the density of mutations averaged across all the ORFs. In Q and R columns, the “x” indicates the genes NE in vitro that could be essential in the infection process in type 1 and type 2 strains, respectively. In column S putative antigenic proteins are also shown fot both types or each type (only type 1 or only type 2). The associated table indicates the results of study of enrichment in COG categories in putative antigenic proteins common to both tyes (1 and 2) by using the Fisher’s test. The attached legend in the table indicates the statistics formula applied for calculating the probability values as well as the values of the different parameters.
(XLSX)

S10 Table Description of non-synonymous mutations found in type 1 and type 2 strains. Columns A to K Description of non-synonymous mutations found in type 1 (A to E) and type 2 (G to K) strains using as reference M129. ID is the description of the non-sysnonymous mutation; ORF is the *M*. *pneumoniae* M129 gene name; Genome position, indicates the location of the mutation in the genome; Protein position, indicates the position in the protein sequence of the changed aa; aa change shows the change (wild type/mutated). Columns M to W, Description of non-synonymous mutations found in type 1 (M to Q) and type 2 (S to W) strains using as reference FH strain.
(XLSX)

S11 Table Study of transcriptomic differences in the sequenced strains. A) ORF name of the studied gene. From B) to I) log 2 of CPKMs (counts per kilobase per million reads) for the two biological replicates of the four studied strains (type 2 strains: 6009 and 2882 strains; type 1a: 5817 and 5954 strains). J) to M) Averages of log2 of CPKMs for the two biological replicates for each one of the studied strains. N) Average of log2 of CPKMs of the four samples corresponding to the two type 2 strains. O) Average of log2 of CPKMs of the four samples corresponding to the two type 1 strains. P) Fold change of log2 CPKMs of type 2 versus type 1 strains. Q) P-value obtained by the T-test. R) Protein name. S) Function associated to the gene. T) COG category: A, membrane proteins of unknown function; C, energy production and conversion, coenzyme metabolism; D, cell division and chromosome partitioning; E, amino acid transport and metabolism; F, nucleotide transport and metabolism, coenzyme; G, carbohydrate transport and metabolism; H, coenzyme metabolism; I, lipid metabolism; J, translation, ribosomal structure, and biogenesis; K, transcription; L, DNA replication, recombination, and repair; M, cell envelope biogenesis, outer membrane; N, cell motility and secretion; O, post-translational modification, protein turnover, chaperones; P, inorganic ion transport and metabolism; R, general function prediction only; S, function unknown; T, signal transduction mechanisms; U, intracellular trafficking, secretion, and vesicular transport; and V, defense mechanisms. The attached table shows the results of the Fisher’s test for the enrichment in COG categories in genes that are differentially expressed between type 1 and type 2 strains.
(XLSX)

S12 Table Quantification of proteins in different strains. In A) to E) are shown the averages of areas obtained for each protein in the first experiment comprising two biological replicates and two technical replicates for each one of the studied strains (four samples for each strain). From G) to K) averages of areas obtained in a second replicate comprising two samples per each strain. M) to O) show the averaged values for each gene in all the strains of type 2 and type 1, respectively, in experiment 1. From Q) to S) averaged values for type 2 and type 1 in experiment 2, respectively.
(XLSX)

S13 Table Study of proteomic fold changes in the sequenced strains. For each protein in column A the value of log2 of the fold change in protein levels is estimated from the two independent experiments comprising 3 biological and 6 technical replicates for each one of the four studied strains (2 type 1 and 2 type 2). Fold changes are obtained after comparing one strain against the others and using the t-test to determine significant changes. The fold change and the corresponding p-value are indicated in consecutive columns for 2882, 6009, 5954 and 5817 for the two independent experiments (Exp1 and Exp2). Also fold changes and p-values after grouping type 1 (5954 and 5817) and type 2 (2882 and 6009) strains are shown in the columns from R to U for the two independent experiments. Different colors in column A show specific changes in different comparative studies considering both experiments: red, specific for 2882 strain; green, specific for 6009 strain; blue, specific for 5494 strain; and yellow, specific of type 2.
(XLSX)
