# Supplementary figures and images for: Comparative “-omics” in Mycoplasma pneumoniae Clinical Isolates Reveals Key Virulence Factors
Source: PLoS One. 2015 Sep 3;10(9):e0137354. doi: 10.1371/journal.pone.0137354 (PMC4559472; doi:10.1371/journal.pone.0137354)

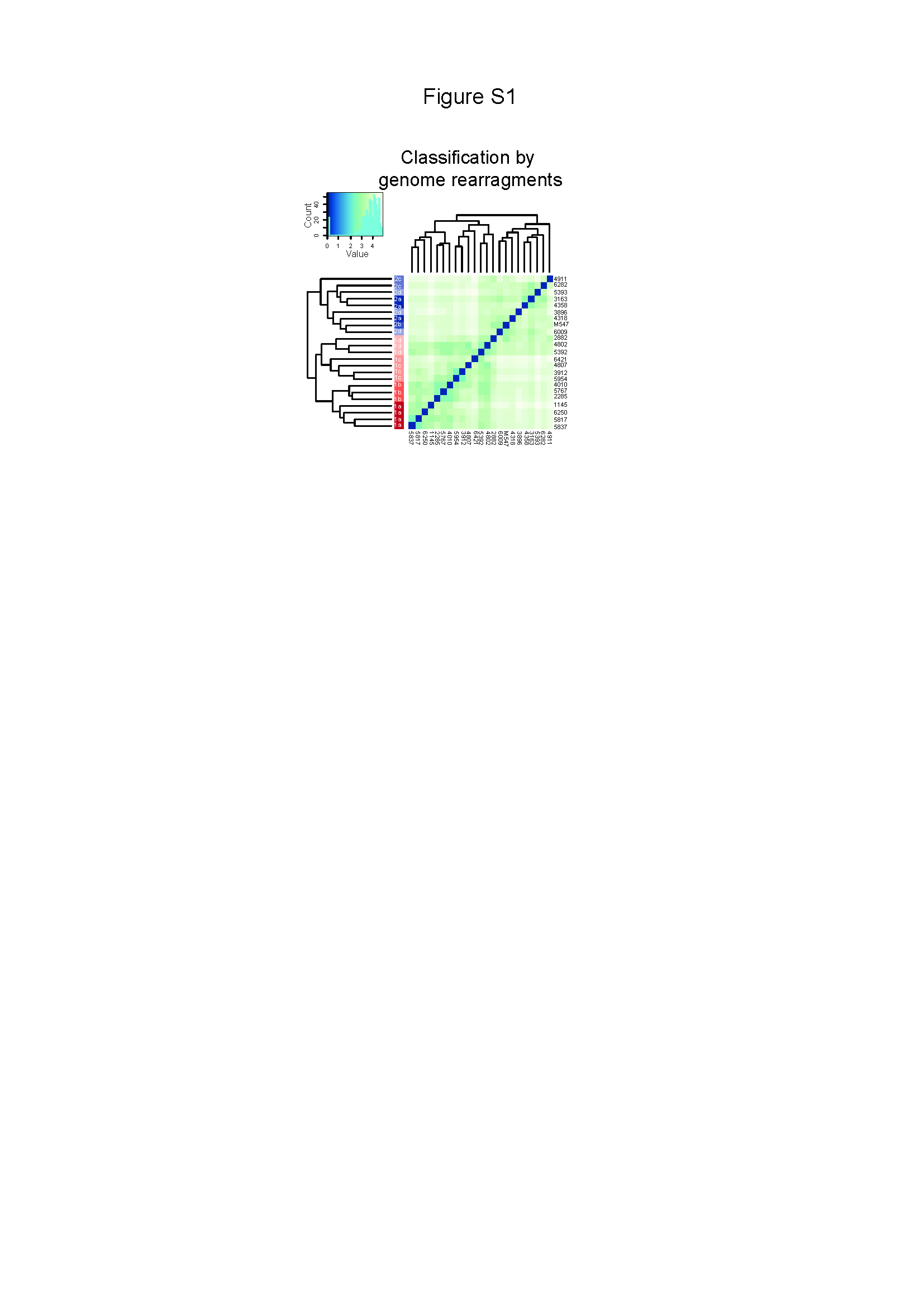

Supplement: S1 Fig — (TIFF) [file pone.0137354.s001.tiff]
